# Indirect effects of Seeking Safety plus sertraline on alcohol use: The mediating role of reductions in posttraumatic stress disorder symptom severity

**DOI:** 10.1002/jts.70031

**Published:** 2025-12-29

**Authors:** Lesia M. Ruglass, Jordan A. Gette, Antonio A. Morgan‐López, Ai Ye, Kathryn Z. Smith, Skye Fitzpatrick, Teresa López‐Castro, Lissette M. Saavedra, Sonya B. Norman, Therese K. Killeen, Sudie E. Back, Denise A. Hien

**Affiliations:** ^1^ Department of Psychology The City College of New York New York New York USA; ^2^ Center of Alcohol & Substance Use Studies Rutgers University–New Brunswick New Brunswick New Jersey USA; ^3^ Department of Psychiatry and Behavioral Sciences University of Kansas Medical Center Kansas City Kansas USA; ^4^ RTI International, Research Triangle Park North Carolina USA; ^5^ Department of Psychology Ludwig Maximilians Universität München Munich Germany; ^6^ Independent Practice Cincinnati Ohio USA; ^7^ Department of Psychology York University Toronto Canada; ^8^ National Center for PTSD White River Junction Vermont USA; ^9^ Department of Psychiatry University of California San Diego, La Jolla California USA; ^10^ Department of Psychiatry and Behavioral Sciences Medical University of South Carolina Charleston South Carolina USA

## Abstract

Posttraumatic stress disorder (PTSD) symptoms and alcohol use frequently co‐occur and are mutually reinforcing. Few studies have examined how changes in PTSD severity influence subsequent changes in alcohol use, particularly in the context of comorbidity treatments. This secondary analysis utilized data from a clinical trial comparing 12 weeks of Seeking Safety plus sertraline (SS+S) versus Seeking Safety plus placebo (SS+P) among individuals (*N* = 69) with co‐occurring PTSD and alcohol use disorder. A mediational analysis, using a latent growth modeling framework from five points throughout treatment, was conducted to examine whether reductions in alcohol use were mediated by treatment‐led (SS+S vs. SS+P) reductions in PTSD severity. Results revealed a nonsignificant treatment effect on PTSD severity reductions over time, Est. = −0.440. The link between PTSD severity reductions and reductions in alcohol use was significant, Est. = 0.644. Despite the nonsignificant path between treatment group and PTSD severity (and in the presence of bias correction for small sample size), the formal test of mediation was significant such that for participants in the SS+S group, alcohol use reductions were fully mediated by reductions in PTSD severity, Est. = −0.283. These findings suggest one of the mechanisms through which SS+S exerts its effects on alcohol use is through reductions in PTSD severity. Clinicians working within the SS treatment platform may consider augmenting their treatment plan with sertraline in collaboration with clients. Future research is necessary to understand how medication and behavioral therapies synergize over a treatment course to improve PTSD and alcohol use.

A diagnosis of posttraumatic stress disorder (PTSD) carries a 2–4‐fold risk of a co‐occurring substance use disorder (SUD) diagnosis (Pietrzak et al., 2011). When PTSD and SUD co‐occur (PTSD+SUD), the severity of each disorder intensifies, resulting in poorer treatment outcomes and an increased risk of relapse compared to individuals with either PTSD or SUD alone (Flanagan et al., 2016; Norman et al., 2018; Sells et al., 2016; Tripp et al., 2019). Theory and empirical studies support the role of PTSD in the maintenance and exacerbation of SUD (Gielen et al., 2016; Hien et al., 2021), with several theoretical models (e.g., self‐medication, susceptibility, and mutual maintenance; Khantzian, 1985, 1997; Smith & Randall, 2012) that explain the mechanisms by which PTSD and SUD mutually impact each other. The self‐medication hypothesis posits that individuals with PTSD may use substances to manage painful emotional states (Khantzian, 2003; Ruglass et al., 2014). Other models include a susceptibility model, in which substance‐related neurobiological impairments enhance the likelihood of developing or worsening PTSD (McCauley et al., 2012; van Dam et al., 2013), and the mutual maintenance model, wherein substances may initially be consumed to ameliorate PTSD symptoms, but their use exacerbates or worsens PTSD symptoms over time (Tripp et al., 2020).

Studies of individuals receiving PTSD+SUD treatments have now begun to test these functional associations and examine whether a change in one set of symptoms influences the second set of symptoms (Back et al., 2006; Hien et al., 2010). For example, Hien and colleagues (2018) applied growth models and cross‐lagged analyses to examine how active substance use during 12 weeks of either an integrated exposure‐based treatment (Concurrent Treatment of PTSD and Substance Use Disorders Using Prolonged Exposure Therapy [COPE; Back et al., 2014)]) or a SUD‐only focused treatment (relapse prevention therapy [RPT]) impacted PTSD symptom severity during treatment and posttreatment in a sample of 82 participants. Overall, the authors found that at posttreatment, a decrease in substance use (going from 1 to 0 days of use) was associated with lower PTSD symptom severity regardless of treatment type, with steeper reductions in PTSD symptoms favoring COPE. This finding supports the hypothesis that continued substance use can lead to a subsequent worsening of PTSD symptoms. Tripp and colleagues (2020) further examined this association in a sample of 107 veterans (89% male) with co‐occurring PTSD and alcohol use disorder (AUD) who were randomized to receive COPE or Seeking Safety (SS; Najavits, 2002), a cognitive behavioral treatment focused on coping skills–building. Utilizing multilevel regression modeling, the results revealed that higher PTSD symptom levels were associated with more subsequent alcohol use. Likewise, more alcohol use during the prior week was associated with higher PTSD symptom severity 1 week later, although this association was not as strong.

In contrast, in a recent application of a time‐lagged model to investigate symptom change dynamics during COPE treatment, Badour et al. (2022) found that PTSD symptoms did not significantly predict next‐week substance use, diverging from preliminary evidence suggesting that PTSD symptom reductions contribute to changes in substance use in integrated PTSD+SUD treatment. The preliminary nature of such analyses, with mixed findings overall, suggests the need for additional research that considers associations between PTSD symptom change and alcohol use in a broader range of interventions, as such effects may be intervention‐specific.

Indeed, the type of treatment delivered may contribute to how and when symptoms change over the course of treatment. For individuals who receive trauma processing treatment (e.g., COPE), the habituation of trauma‐related fear and distress may diminish avoidant coping, such as substance use (Badour et al., 2017; Rauch & Foa, 2006). Therefore, reductions in PTSD symptoms may be likely to precede reductions in alcohol or substance use in trauma processing interventions. In a coping skills model, such as SS, changes in coping skills may lead to a simultaneous reduction in both PTSD and substance use symptoms. Many studies examining the sequencing of change between PTSD and AUD targets have focused on trauma processing treatments.

A small number of studies suggest that enhancing PTSD symptom reductions by combining psychotherapies with medications that can target SUD or PTSD may be a promising strategy (Hien et al., 2023). For example, one RCT (Hien et al., 2015) examined the combination of SS with sertraline, a selective serotonin reuptake inhibitor medication approved in the United States for the treatment of PTSD (Hoskins et al., 2021; Schwartz & Rothbaum, 2002), and found clinically significant reductions in both PTSD and AUD severity at posttreatment that were sustained through 12‐month follow‐up, with larger PTSD symptom improvements observed across all follow‐up assessments in the SS plus sertraline (SS+S) condition compared to the SS plus placebo (SS+P) condition (Hien et al., 2015). Although these findings were promising and indicated that enhanced PTSD improvement occurred for individuals assigned to SS+S, the focus on pretreatment‐to‐posttreatment changes in symptom levels limits understanding of how participants’ symptoms were changing over time (Laurenceau et al., 2007).

Given that the use of adjunctive medications may significantly influence the impact and mechanisms of a PTSD+SUD intervention, the specific interplay of PTSD and SUD symptom change over the course of treatment may also be distinct when medications are included in PTSD+SUD interventions. However, all studies examining temporality in symptom change that have been described thus far have focused on behavioral therapies; it remains unclear whether these results are generalizable to treatments that combine behavioral therapies and pharmacotherapies. Moreover, although the analytic models utilized in these studies parsed the temporal sequencing of changes in PTSD and SUD, they could not simultaneously determine how and whether a change in one set of symptoms was associated with changes in the other over time, which would address natural growth processes. Thus, additional studies that use alternative analytic models are needed to better address the causal ordering question alongside modeling growth trajectories of changes in each symptom set.

To address some of these concerns, we conducted a secondary analysis of the data from Hien et al.’s (2015) RCT to examine whether treatment type (SS+S vs. SS+P) impacted the overall rate of change over time (i.e., growth curves) on the two constructs or had mediation effects on days of alcohol use via direct effects on PTSD. We expected larger reductions in PTSD for SS+S versus SS+P. In turn, we expected that changes in PTSD symptoms would be associated with later reductions in alcohol use (Hien et al., 2010). The findings from this analysis could provide new information to help inform clinicians in their decision‐making with regard to treatment planning, particularly when medications are involved.

## METHOD

### Participants

Data for these analyses were from Hien et al.’s (2015) completed RCT that tested the efficacy of combining sertraline with SS in a sample of 69 women with co‐occurring PTSD and AUD. Data were collected from 2006 to 2012. See Hien et al. (2015) for complete details on the procedures. All procedures were reviewed and approved by the Institutional Review Boards of The New York Psychiatric Institute and The City College of New York. All participants provided written informed consent. See Table 1 for sample demographic and diagnostic characteristics.

**TABLE 1:** Baseline demographic and diagnostic characteristics, by treatment group

	Seeking Safety + sertraline		Seeking Safety + placebo	
	(*n* = 32)		(*n* = 37)	
Characteristic	*n*	%	*n*	%
Women	26	81.3	30	81.1
Race/ethnicity				
African American	16	50.0	25	67.6
European American	10	31.3	6	16.2
Latina/o	3	9.4	4	10.8
Other	3	9.4	2	5.4
Marital status				
Married	9	28.1	5	13.5
Single	17	53.1	25	67.6
Divorced/separated/widowed	6	18.8	7	18.9
Employment				
Employed	23	71.9	30	81.1
Unemployed	8	25.0	4	10.8
Student/retired/disabled	1	3.1	3	8.1
Past 7‐day abstinence rate	3	9.7	4	10.8
*DSM‐IV* Alcohol dependence	28	87.5	33	89.2
*DSM‐IV* Alcohol abuse	3	9.4	0	0
Early‐onset AUD	13	40.6	16	48.5
Drug dependence				
Cannabis	5	15.6	3	8.1
Cocaine	8	25.0	13	35.1
Comorbid AUD and SUD	16	50.0	22	59.5
Lifetime traumatic experiences^a^				
Child physical	14	43.3	18	48.5
Adult physical	16	50.0	16	42.4
Child sexual	12	36.7	15	41.2
Adult sexual	12	36.7	13	35.3
Transportation accident	19	60.0	27	73.5
Life‐threatening illness	7	23.3	8	20.6
Exposed to violent death	14	43.3	10	26.5
Current major depression	20	62.5	22	59.5

	M	S*D*	M	S*D*
Age (years)	42.2	9.8	42.5	8.5
Education (years)	13.7	3.1	13.0	2.0
Age at PTSD onset	28.1	14.4	22.8	13.5
CAPS severity, total	65.8	19.4	59.0	19.2
Past–7 day DDD	6.8	5.1	6.9	4.7
Past–7 day HDD	3.3	2.2	2.9	2.4
Prior alcohol treatment episodes	1.1	1.9	1.6	4.3

*Note*: *N*  = 69. *DSM‐IV* = *Diagnostic and Statistical Manual of Mental Disorders* (4th ed.); AUD = alcohol use disorder (*DSM‐IV* abuse or dependence); SUD = substance use disorder (*DSM‐IV* abuse or dependence); PTSD = posttraumatic stress disorder; CAPS = Clinician Administered PTSD Scale; DDD = drinks per drinking day; HDD = heavy drinking day (men: ≥ 4 four drinks, women: ≥ 4 drinks).

^a^ No participants endorsed a natural disaster event.

Inclusion criteria were (a) meeting the *Diagnostic and Statistical Manual of Mental Disorders* (4th ed., text rev.; *DSM‐IV‐TR*; American Psychiatric Association [APA], 2000) criteria for full PTSD or subthreshold PTSD (Grubaugh et al., 2005), defined as meeting Criteria A (exposure to a traumatic stressor), B (reexperiencing symptoms), E (symptom duration of at least 1 month), and F (significant distress or impairment of functioning) and either Criterion C (avoidance and/or numbing symptoms) or Criterion D (increased arousal symptoms) or (b) meeting the *DSM‐IV‐TR* criteria for current alcohol abuse or dependence. Individuals who did not meet the criteria for alcohol abuse or dependence were also eligible if they reported alcohol misuse in the past 90 days, defined as either hazardous drinking or binge drinking per the National Institute on Alcohol Abuse and Alcoholism (NIAAA) criteria (NIAAA, 2025).

### Procedure

As described in Hien et al. (2015), individuals who completed the prerandomization requirements phase were accepted into the study and randomly assigned to one of the two treatment conditions: SS+S (*n* = 32) or SS+P (*n* = 37). Participants completed weekly assessments during the 12‐week treatment and were reassessed at 1‐week, 6‐month, and 12‐month posttreatment follow‐ups. There were no differences in attrition by treatment condition (Hien et al., 2015). Average session attendance rates for SS sessions were equivalent across treatment conditions.

#### Intervention

##### Cognitive behavioral therapy

SS (Najavits, 2002) is a manualized, 12‐week intervention that utilizes cognitive behavioral strategies to assist participants in attaining abstinence from substances and decreasing trauma‐related symptoms and PTSD. The intervention was delivered in a 12‐session, 60‐min weekly individual format by eight experienced (PhD‐ or licensed clinical social worker–level) research therapists who underwent rigorous training in the SS protocol. Participants had up to 14 weeks to complete all 12 sessions. The content of each session was structured to provide a theme relevant to both substance use disorders and PTSD, and a specific cognitive behavioral therapy skill to learn.

##### Medication

Matching capsules contained either sertraline or a placebo, as well as riboflavin, which was used to assess medication adherence. Compliance was also monitored by pill count. Participants who received sertraline started at 50 mg daily and were titrated up to a maximum of 200 mg daily over a 2‐week period, as per standard protocols, to determine therapeutic efficacy by the project psychiatrists. Participants continued their therapeutic sertraline dose until the end of the trial and were tapered after unblinding. Responders were offered the option to remain on medication after the end of the trial.

#### Reliability training

During the original trial, independent assessors (IAs), who held at least a master's degree or a PhD degree and had clinical experience, completed a 1‐day, expert‐led training session on administering and scoring the Structured Clinical Interview for *DSM‐IV‐TR* Axis I Disorders (SCID‐*DSM‐IV*; First et al., 2002) and the Clinician‐Administered PTSD Scale for the *DSM‐IV* (CAPS; Blake et al., 1995). The IAs had weekly calls with the lead team to maintain competency and interrater reliability on the measures. The reliability of diagnoses was assessed by reviewing 10% of all baseline assessments and 10% of all follow‐up assessments. Kappa values for the SCID diagnosis and PTSD measures were computed between the IA and expert raters. Raters were expected to have a .70 level of agreement; if agreement levels fell below .70, the supervisor conducted joint rated interviews with the IA until a .70 level was achieved in three consecutive interviews. IAs were blind to the treatment condition for all participants for the entirety of the study.

### Measures

#### *DSM‐IV‐TR* diagnoses

The SCID‐I (First et al., 2002) was administered at baseline and follow‐up assessments to measure current alcohol and other drug use disorder (AOD) diagnoses, age of AOD onset, and the presence of any other current or past mood disorder (e.g., major depressive disorder, dysthymic disorder). AOD diagnoses were considered current if the diagnostic criteria were met in the prior 6 months. The SCID‐I has demonstrated high interrater reliability.

#### Sociodemographic characteristics

Age, race/ethnicity, education (years), relationship status, and employment status were collected at the baseline assessment.

#### PTSD diagnosis

The CAPS (Blake et al., 1995), a 30‐item structured clinical interview, was used to assess the frequency and intensity of *DSM*‐*IV* PTSD symptoms, impairments in social and occupational functioning, diagnosis, and overall symptom severity. The scale consists of three symptom cluster subscales: Reexperiencing, Avoidance/Numbing, and Hyperarousal. Frequency and intensity scores for each subscale are summed to obtain an overall total score. The CAPS‐IV has demonstrated strong reliability and validity across multiple studies (Weathers et al., 2001). Data for this study showed good‐to‐excellent internal consistency across baseline and all follow‐up assessments, Cronbach's αs = .86–.92.

#### PTSD symptoms

The Modified Posttraumatic Stress Disorder Symptom Scale–Self Report (MPSS‐SR; Falsetti et al., 1993) is a 17‐item, self‐report instrument that is used to assess the frequency and severity of *DSM‐IV‐TR* PTSD symptoms. Participants completed the MPSS‐SR at baseline, weekly during treatment, and at all posttreatment follow‐up assessments. Psychometric studies of the MPSS‐SR with similar comorbid PTSD+SUD treatment samples have demonstrated high concurrent validity with the CAPS and suggest it is a reliable tool for monitoring PTSD symptoms (Ruglass et al., 2014). In the current sample, the MPSS‐SR demonstrated excellent internal consistency at baseline, Cronbach's α = .93.

#### Alcohol use

A single item from the Substance Use Inventory (SUI; Weiss et al., 1995) was used to assess the self‐reported frequency of days of alcohol use in the previous 7 days. The SUI was administered at baseline, weekly during the trial, and at all posttreatment follow‐up assessments.

### Data analysis

#### PTSD calibration

As outlined, trained assessors administered the CAPS‐IV‐TR at baseline to establish a PTSD diagnosis and baseline symptom severity, and the self‐report MPSS‐SR was administered weekly throughout treatment. However, as possible misalignment in symptom reporting due to administration method and bias, wording difference, response scaling, and patient characteristics (Morgan‐López et al., 2020) could lead to differences between measures (e.g., CAPS vs. MPSS‐SR), we calibrated the MPSS‐SR to the CAPS before analysis. Because all participants were given both the CAPS and the MPSS‐SR, common persons calibration (Chen et al., 2009) was used to estimate a latent PTSD severity score at each assessment point via the following steps in M*plus* (Version 8; Muthén & Muthén, 2017). First, a unidimensional nonlinear factor analysis model was estimated using only the CAPS items to create a single‐factor model of PTSD, with factor loadings and item thresholds to be used in the following calibration step. Next, a model was estimated in which the MPSS‐SR items were added to the single‐factor model, with their item parameters freely estimated and the item parameters from the CAPS items fixed to the values from the previous step. This step allowed for the MPSS‐SR items to be calibrated to the CAPS in the same manner that new items for educational assessments (e.g., the SAT or GRE) are calibrated to old versions of tests (e.g., Dorans, 2007; Morgan‐López et al., 2020). The factor scores from this step were then used in all subsequent models. See Supplementary Tables S1 and S2 for item parameters for both measures.

#### Model estimation

A mediation analysis using the latent growth modeling framework (Cheong et al., 2003), with full information maximum likelihood (FIML) estimation, was conducted to examine whether associations between treatment group (i.e., SS+S vs. SS+P) and reductions in alcohol use were mediated by reductions in PTSD symptom severity. FIML estimation uses all available data and assumes that the probability of missingness is related to variables that are observed but (conditionally) unrelated to variables that are missing (i.e., missing at random; Schafer & Graham, 2002)

The mediation analysis was conducted using five of the total assessment points: baseline (T1), midtreatment (T9), end of treatment (T16), 6 months posttreatment (T17), and 12 months posttreatment (T18). Given the moderate sample size, this subset of assessment points was selected to largely reflect the time points utilized in the primary outcomes paper (Hien et al., 2015). Using a longitudinal framework with nonlinear parallel growth processes (Cheong et al., 2003; Harring et al., 2021), we examined paths from treatment type to slope for changes in PTSD severity (Path *a*), from changes in PTSD severity to drinking days (Path *b*), and the indirect effect of treatment type on drinking days via changes in PTSD symptom severity (MacKinnon et al., 2002). This model fit the data well, comparative fit index (CFI) = .950, root mean square error of approximation (RMSEA) = .040.

Due to the small sample size, general nonnormality in the sampling distribution of the mediation effect (MacKinnon, in press; MacKinnon et al., 2002), and nonnormality in the distribution of the PTSD and alcohol use outcome variables (Kelley, 2005; Saavedra et al., 2023), mediated effect confidence intervals were estimated using bias‐corrected bootstrapping procedures (Efron & Tibshirani, 1994) via the M*plus* CINTERVAL(BCBOOT) command, with 5,000 bootstrap resamples to estimate the empirical confidence intervals.

## RESULTS

Means, standard deviations, and correlations among study variables are presented in Table 2. The treatment effect favoring SS+S (vs. SS+P) on reductions in PTSD severity over time was nonsignificant, Path *a* estimate (Est.) = −0.444, 95% CI [‐1.041, 0.002], whereas the link between reductions in PTSD severity and reductions in alcohol severity was significant, Path *b* Est. = 0.644, 95% CI [0.041, 1.776]. The direct effect of SS+S (Path *c*) on alcohol use was nonsignificant, *p* = .683. Although Path *a* was nonsignificant, and in the presence of bias correction for small sample size (MacKinnon et al., 2004), the formal test of mediation was significant, Path *ab* Est. = −0.283, 95% CI [‐1.199, −0.032], such that for participants in the SS+S group, reductions in alcohol use were fully mediated by reductions in PTSD symptom severity. The total effect—the effect of SS+S versus SS+P on changes in substance use, without PTSD as a mediator—was nonsignificant, *p* = .429, consistent with Hien et al.’s (2015) findings (see Table 3).

**TABLE 2:** Correlations among latent drinking and posttraumatic stress disorder (PTSD) symptom variables

	T1	T9	T16	T17	T8	T1	T9	T16	T17	T18			
Variable	DD	DD	DD	DD	DD	PTSD	PTSD	PTSD	PTSD	PTSD	TX	*M*	*SD*
T1 DD	–	.094	.244	.432	.373	.049	−.077	−.193	−.021	−.011	.127	3.95	2.21
T9 DD		–	.471	−.027	.383	.202	.344	.270	.125	.216	.081	2.03	2.22
T16 DD			–	.488	.700	.267	.319	.456	.416	.434	.085	1.51	1.95
T17 DD				–	.357	.210	.248	.131	.392	.319	.095	1.39	1.94
T18 DD					–	.267	−.193	.131	−.027	−.011	.095	1.03	1.62
T1 PTSD						–	.315	.252	.428	.212	.126	1.54	0.54
T9 PTSD							–	.465	.652	.611	−.122	0.57	1.33
T16 PTSD								–	.416	.648	.028	0.55	1.25
T17 PTSD									–	.778	−.244	0.64	0.92
T18 PTSD										–	−.236	0.45	1.21
TX											–	0.48	0.50

*Note*: Treatment was coded such that 0 = Seeking Safety plus placebo and 1 = Seeking Safety plus sertraline. DD = drinking days; TX = treatment; T1 = baseline; T9 = midtreatment; T16 = end of treatment; T17 = 6‐month follow‐up; T18 = 12‐month follow‐up.

**TABLE 3:** Mediation effects of intervention condition on days of alcohol use as transmitted through changes in posttraumatic stress disorder (PTSD) symptom severity

Path	Description	Estimate	95% CI	*SE*
Path *a*	Treatment predicting PTSD slope	−0.440	[‐1.042, 0.003]	0.266
Path *b* ^a^	PTSD slope predicting drinking slope	0.644	[0.041, 1.766]	0.412
Path *c*	Total effect of treatment predicting drinking slope	0.238	[−0.248, 0.967]	0.301
Path *c*’	Direct effect of treatment predicting drinking slope	0.230	[−0.702, 1.613]	0.563
Path *ab* ^a^	Indirect path treatment predicting drinking slope via PTSD	−0.283	[−1.199, −0.003]	0.259

*Note*: Path *a* is the treatment‐to‐mediator path, Path *b* is the mediator‐to‐outcome path, Path *c* is the total effect of treatment predicting drinking prior to accounting for mediation effects, Path *c*’ is the direct effect of treatment predicting drinking after the inclusion of mediation effects, and Path *ab* is the indirect effect of treatment to outcome via the mediator. CI = confidence interval.

^a^ Significant path.

## DISCUSSION

This manuscript aimed to identify whether treatment type (SS+S vs. SS+P) impacted the overall rate of change over time on PTSD symptoms and alcohol use or had mediation effects on alcohol trajectories via direct effects on PTSD. Overall, the findings support time‐varying changes in PTSD symptom severity, alcohol use reductions during treatment, and a mediation effect over the duration of treatment and follow‐up. Using a mediation model under a latent growth modeling framework, we found that the effects of SS+S on alcohol trajectories were fully mediated by changes in PTSD symptom severity trajectories. The findings of PTSD‐targeted medication effects on alcohol use over time are consistent with other recent work showing that the indirect effects of PTSD‐targeted medications influence substance use through changes in PTSD symptoms (Hien et al., 2025). This mediation finding aligns with a treatment model that addresses self‐medication by reducing PTSD symptom severity, which, in turn, is expected to mitigate alcohol use outcomes (Khantzian, 1985). Moreover, the medication findings suggest that clinicians working within the SS therapy platform should consider a collaborative discussion with PTSD+SUD clients regarding augmenting their treatment plans with sertraline. The impact of adding sertraline to SS on reducing PTSD severity may be a key pathway to larger reductions in alcohol use throughout treatment. Although the results of a recent integrative data analysis of 36 randomized controlled trials of psychotherapy, medication, and combination treatments for co‐occurring PTSD+SUD found that trauma‐focused treatments, such as COPE, are more robust in reducing PTSD than other types of PTSD or SUD treatments (Hien et al., 2023), which, in turn contributed to improved alcohol use outcomes (Hien et al., in press), our findings suggest a non–trauma‐focused or coping skills–based treatment, such as SS, augmented with sertraline may provide similar benefits in reducing clients’ PTSD at a level that is associated with reduced alcohol use. Sertraline may contribute to enhanced PTSD severity reductions by reducing mood dysregulation and supporting the acquisition of skills delivered in SS. However, given the variability in dosing of and response to sertraline for any given client, the enhanced benefit of sertraline may not be evident until the end of treatment and beyond. Thus, ongoing weekly monitoring of PTSD and alcohol use may provide helpful information in pinpointing the timing when reductions in PTSD symptom severity may synergistically contribute to improvements in alcohol use. Finally, the findings support the notion that targeting alcohol use is not sufficient to reduce PTSD symptom severity, which, if not treated, remains a salient trigger for substance use.

Several limitations should be noted. Given that the sample was predominantly women with PTSD and alcohol misuse or AUD, the findings may not be generalizable to other populations and other types of substances. Since data collection, the diagnostic criteria for both PTSD and AUD have changed (Pomeroy & Anderson, 2013); thus, the findings may not fully be generalizable to the current diagnostic criteria and should be replicated with samples diagnosed using the current diagnostic criteria (i.e., *DSM‐5‐TR*; APA, 2022). The small sample size may have also influenced the findings. We were unable to test other purported mechanisms of change because we did not have the specific measures with which to do so, beyond the evaluation of the functional association between PTSD and alcohol use. Future studies are recommended to replicate findings with more diverse samples (e.g., stratified by sex) and other types of substances (e.g., cocaine, cannabis).

Despite these limitations, this study is one of the first to examine the functional association between PTSD and days of alcohol use in a sample receiving a combination of psychotherapy plus medication. The findings highlight the benefits of augmenting SS with sertraline to enhance reductions in PTSD symptom severity over the course of treatment, which, in turn, are associated with reductions in alcohol use over time. And although studies suggest that trauma‐focused treatments (e.g., COPE) may be more robust in reducing PTSD symptoms, our results suggest a coping skills–based or non–trauma‐focused platform, such as SS +S, can positively impact PTSD trajectories over time, leading to alcohol use reductions and, thus, should be included as a treatment option for individuals with co‐occurring PTSD+SUD.

## AUTHOR NOTE

This study was supported by the National Institute on Alcohol Abuse and Alcoholism (R01AA014341).

## AUTHOR CONTRIBUTIONS

Ruglass, L.M.: Conceptualization; Writing ‐ original draft; Writing ‐ review & editing; Supervision. Gette, J.: Formal analysis; Writing ‐ original draft; Writing ‐ review & editing. Morgan‐López, A.A.: Conceptualization; Writing ‐ original draft; Writing ‐ review & editing; Formal analysis; Supervision. Ye A.: Formal analysis; Writing ‐ review & editing; Writing ‐ original draft. Smith, K.M.: Conceptualization; Writing ‐ original draft; Writing ‐ review & editing. Fitzpatrick, S.: Writing ‐ original draft; Writing ‐ review & editing. Lopez‐Castro, T.: Writing ‐ original draft; Writing ‐ review & editing. Saavedra, L.: Writing ‐ original draft; Writing ‐ review & editing. Norman, S.B.: Writing ‐ original draft; Writing ‐ review & editing. Killeen, T.: Writing ‐ original draft; Writing ‐ review & editing. Back, S.E.: Writing ‐ original draft; Writing ‐ review & editing. Hien, D.A.: Conceptualization; Funding acquisition; Writing ‐ review & editing; Writing ‐ original draft; Supervision.

## OPEN PRACTICES STATEMENT

The clinical trial in this study is registered at ClinicalTrials.gov (NCT00262223). The data that support the findings of this study are available from the principal investigator upon reasonable request at denise.hien@rutgers.edu.

The authors have no known conflict of interest to disclose.

## Supporting information



SUPPORITING INFORMATION
