# Supplementary material for: Indirect effects of Seeking Safety plus sertraline on alcohol use: The mediating role of reductions in posttraumatic stress disorder symptom severity
Source: J Trauma Stress. Author manuscript; Available in PMC 2026 Apr 15. (PMC13044387; doi:10.1002/jts.70031)
Supplement: Supplemental Tables [file NIHMS2150789-supplement-Supplemental_Tables.docx]

Supplementary Table S1

Factor Loadings of the Baseline Modified Posttraumatic Stress Disorder Symptom Scale – Self-Report (MPSS-SR) Prior to Calibration

| MPSS-SR  Item | Loading | Threshold  (0, 1) | Threshold  (1, 2) | Threshold  (2, 3) | Threshold  (3, 4) |
| --- | --- | --- | --- | --- | --- |
| 1F: Intrusive Thoughts | 2.655 | -1.748 | 0.657 | 3.075 | - |
| 1S: Intrusive Thoughts | 2.466 | -1.707 | 0.165 | 2.204 | 3.876 |
| 2F: Nightmares | 1.875 | -0.369 | 1.136 | 2.833 | - |
| 2S: Nightmares | 1.835 | -0.370 | 0.796 | 2.070 | 3.248 |
| 3F: Flashbacks | 2.047 | -0.117 | 1.427 | 3.115 | - |
| 3S: Flashbacks | 2.198 | -0.145 | 1.097 | 2.398 | 3.970 |
| 4F: Emotional Reactivity | 2.861 | -2.355 | 0.149 | 2.792 | - |
| 4S: Emotional Reactivity | 2.767 | -2.298 | -0.103 | 1.664 | 3.370 |
| 5F: Internal Avoidance | 2.267 | -1.663 | -0.178 | 1.394 | - |
| 5S: Internal Avoidance | 2.141 | -1.762 | -0.247 | 1.000 | 2.452 |
| 6F: External Avoidance | 2.515 | -1.443 | -0.158 | 1.338 | - |
| 6S: External Avoidance | 2.482 | -1.481 | -0.226 | 0.990 | 2.482 |
| 7F: Recall Difficulty | 1.456 | 0.999 | 1.726 | 2.646 | - |
| 7S: Recall Difficulty | 1.451 | 0.949 | 1.600 | 2.270 | 3.041 |
| 8F: Loss of Interest | 2.637 | -1.142 | 0.601 | 2.376 | - |
| 8S: Loss of Interest | 2.593 | -1.052 | 0.526 | 1.675 | 3.388 |
| 9F: Socially Distant | 2.296 | -0.782 | 0.348 | 1.964 | - |
| 9S: Socially Distant | 2.280 | -0.833 | 0.272 | 1.518 | 3.027 |
| 10F: Lack Positive Emotions | 2.226 | -0.121 | 0.950 | 2.441 | - |
| 10S: Lack Positive Emotions | 2.359 | -0.123 | 0.903 | 2.202 | 3.412 |
| 11F: Foreshortened Future | 1.798 | -0.035 | 1.000 | 2.065 | - |
| 11S: Foreshortened Future | 1.789 | -0.060 | 0.837 | 1.805 | 2.716 |
| 12F: Sleep Difficulty | 2.317 | -1.436 | -0.289 | 1.068 | - |
| 12S: Sleep Difficulty | 2.334 | -1.426 | -0.278 | 0.690 | 1.833 |
| 13F: Irritability | 1.896 | -0.626 | 0.844 | 2.537 | - |
| 13S: Irritability | 1.876 | -0.551 | 0.797 | 2.061 | 3.358 |
| 14F: Concentration | 2.455 | -1.146 | 0.676 | 2.330 | - |
| 14S: Concentration | 2.428 | -1.053 | 0.732 | 2.039 | 3.383 |
| 15F: Overly Alert | 2.180 | -0.411 | 0.807 | 2.112 | - |
| 15S: Overly Alert | 1.969 | -0.474 | 0.738 | 1.702 | 2.801 |
| 16F: Easily Startled | 2.030 | -0.661 | 0.767 | 2.257 | - |
| 16S: Easily Startled | 1.944 | -0.830 | 0.778 | 1.952 | 3.096 |
| 17F: Physical Reactivity | 2.626 | -0.426 | 1.212 | 3.059 | - |
| 17S: Physical Reactivity | 2.404 | -0.464 | 1.072 | 2.332 | 3.431 |

*Note.* F = frequency item, S = severity item. Frequency items are scored on a 4-point scale and severity items are scored on a 5-point scale The MPSS-SR was administered at baseline, weekly throughout the treatment course, and at all follow-up appointments. The baseline scores were used for calibration. Calibrated scores were used in the subsequent analyses in the main text.

Supplementary Table S2

Factor Loadings of the Baseline Modified Posttraumatic Stress Disorder Symptom Scale – Self-Report (MPSS-SR) Calibrated to the Baseline Clinician Administered PTSD Scale for DSM-IV (CAPS-IV)

| Item | Loading | Threshold  (0, 1) | Threshold  (1, 2) | Threshold  (2, 3) | Threshold  (3, 4) |
| --- | --- | --- | --- | --- | --- |
| **MPSS-SR Items** | | | | | |
| 1F: Intrusive Thoughts | 2.441 | 0.482 | 2.854 | 5.204 | - |
| 1S: Intrusive Thoughts | 2.249 | 0.348 | 2.190 | 4.176 | 5.778 |
| 2F: Nightmares | 1.736 | 1.203 | 2.693 | 4.348 | - |
| 2S: Nightmares | 1.701 | 1.168 | 2.324 | 3.573 | 4.715 |
| 3F: Flashbacks | 1.860 | 1.569 | 3.071 | 4.698 | - |
| 3S: Flashbacks | 2.015 | 1.683 | 2.899 | 4.164 | 5.665 |
| 4F: Emotional Reactivity | 2.633 | 0.047 | 2.531 | 5.104 | - |
| 4S: Emotional Reactivity | 2.564 | 0.021 | 2.216 | 3.956 | 5.614 |
| 5F: Internal Avoidance | 2.043 | 0.217 | 1.673 | 3.203 | - |
| 5S: Internal Avoidance | 1.943 | 0.017 | 1.509 | 2.726 | 4.138 |
| 6F: External Avoidance | 2.305 | 0.662 | 1.927 | 3.395 | - |
| 6S: External Avoidance | 2.283 | 0.600 | 1.839 | 3.027 | 4.484 |
| 7F: Recall Difficulty | 1.374 | 2.237 | 2.959 | 3.865 | - |
| 7S: Recall Difficulty | 1.378 | 2.192 | 2.841 | 3.503 | 4.260 |
| 8F: Loss of Interest | 2.452 | 1.090 | 2.811 | 4.559 | - |
| 8S: Loss of Interest | 2.435 | 1.162 | 2.726 | 3.861 | 5.546 |
| 9F: Socially Distant | 2.150 | 1.172 | 2.293 | 3.891 | - |
| 9S: Socially Distant | 2.153 | 1.118 | 2.219 | 3.457 | 4.948 |
| 10F: Lack Positive Emotions | 2.084 | 1.767 | 2.825 | 4.295 | - |
| 10S: Lack Positive Emotions | 2.194 | 1.869 | 2.876 | 4.143 | 5.321 |
| 11F: Foreshortened Future | 1.704 | 1.503 | 2.537 | 3.593 | - |
| 11S: Foreshortened Future | 1.697 | 1.474 | 2.371 | 3.330 | 4.224 |
| 12F: Sleep Difficulty | 2.131 | 0.508 | 1.647 | 2.982 | - |
| 12S: Sleep Difficulty | 2.169 | 0.545 | 1.688 | 2.646 | 3.771 |
| 13F: Irritability | 1.814 | 1.006 | 2.488 | 4.178 | - |
| 13S: Irritability | 1.783 | 1.056 | 2.411 | 3.671 | 4.937 |
| 14F: Concentration | 2.303 | 0.946 | 2.759 | 4.397 | - |
| 14S: Concentration | 2.291 | 1.022 | 2.808 | 4.112 | 5.430 |
| 15F: Overly Alert | 2.059 | 1.459 | 2.676 | 3.966 | - |
| 15S: Overly Alert | 1.858 | 1.206 | 2.418 | 3.370 | 4.450 |
| 16F: Easily Startled | 1.890 | 1.055 | 2.475 | 3.938 | - |
| 16S: Easily Startled | 1.807 | 0.809 | 2.410 | 3.564 | 4.675 |
| 17F: Physical Reactivity | 2.427 | 1.786 | 3.391 | 5.184 | - |
| 17S: Physical Reactivity | 2.214 | 1.548 | 3.059 | 4.282 | 5.341 |
| **CAPS-IV Items** | | | | | |
| B1F: Intrusive Thoughts | 1.566 | 0.584 | 1.595 | 2.892 | 3.340 |
| B1S: Intrusive Thoughts | 1.785 | 0.720 | 1.632 | 3.252 | 5.178 |
| B2F: Nightmares | 1.480 | 1.513 | 2.563 | 3.556 | 4.388 |
| B2S: Nightmares | 1.721 | 1.669 | 2.066 | 2.451 | 4.061 |
| B3F: Flashbacks | 1.380 | 2.430 | 3.197 | 4.140 | 5.152 |
| B3S: Flashbacks | 1.410 | 2.501 | 2.907 | 3.698 | 5.610 |
| B4F: Emotional Reactivity | 1.905 | -0.161 | 2.232 | 3.392 | 4.767 |
| B4S: Emotional Reactivity | 1.859 | -0.141 | 1.204 | 3.132 | 5.285 |
| B5F: Internal Avoidance | 1.573 | 1.155 | 2.734 | 3.744 | 5.627 |
| B5S: Internal Avoidance | 1.506 | 1.039 | 1.428 | 2.648 | 3.788 |
| C1F: External Avoidance | 1.772 | 0.640 | 1.453 | 2.648 | 3.788 |
| C1S: External Avoidance | 2.125 | 0.640 | 1.453 | 2.103 | 2.685 |
| C2F: Recall Difficulty | 1.504 | 0.948 | 1.539 | 3.070 | 6.222 |
| C2S: Recall Difficulty | 1.862 | 1.094 | 1.813 | 2.358 | 2.965 |
| C3F: Loss of Interest | 0.853 | 1.420 | 2.294 | 3.336 | 5.411 |
| C3S: Loss of Interest | 0.714 | 2.566 | 2.866 | 3.558 | 4.627 |
| C4F: Socially Distant | 3.281 | 2.464 | 2.737 | 3.188 | 4.001 |
| C4S: Socially Distant | 3.314 | 3.773 | 4.497 | 5.143 | 6.437 |
| C5F: Lack Positive Emotions | 2.259 | 3.829 | 4.548 | 5.759 | 7.302 |
| C5S: Lack Positive Emotions | 2.353 | 1.914 | 2.323 | 3.080 | 3.995 |
| C6F: Foreshortened Future | 1.965 | 2.135 | 2.572 | 3.678 | 6.460 |
| C6S: Foreshortened Future | 1.796 | 2.286 | 2.671 | 3.537 | 4.452 |
| C7F: Sleep Difficulty | 1.557 | 2.192 | 2.571 | 3.810 | 7.515 |
| C7S: Sleep Difficulty | 1.565 | 3.601 | 3.990 | 4.529 | 5.123 |
| D1F: Irritability | 1.869 | 3.596 | 3.841 | 5.249 | 6.357 |
| D1S: Irritability | 1.733 | 1.058 | 1.542 | 1.956 | 2.909 |
| D2F: Concentration | 1.322 | 0.998 | 1.426 | 2.397 | 3.521 |
| D2S: Concentration | 1.159 | 0.874 | 1.560 | 2.519 | 3.425 |
| D3F: Overly Alert | 2.703 | 0.761 | 1.209 | 2.321 | 4.725 |
| D3S: Overly Alert | 2.383 | 3.248 | 3.973 | 5.270 | 6.420 |
| D4F: Easily Startled | 0.797 | 2.874 | 3.597 | 5.591 | 8.053 |
| D4S: Easily Startled | 0.926 | 0.308 | 0.673 | 1.150 | 1.640 |
| D5F: Physical Reactivity | 1.159 | 0.443 | 0.905 | 2.477 | 4.466 |
| D5S: Physical Reactivity | 1.164 | 1.361 | 1.495 | 3.397 | 4.327 |

*Note.* F = frequency item, S = severity item. MPSS-SR frequency items are scored on a 4-point scale and severity items are scored on a 5-point scale. The CAPS-IV was administered at baseline and the MPSS-SR was administered at baseline, weekly throughout the treatment course, and at all follow-up appointments. The baseline scores were used for calibration. Calibrated scores were used in the subsequent analyses in the main text.
